# Hepatoprotective Effect of Albumin Peptide Fractions from Corn Germ Meal against Alcohol-Induced Acute Liver Injury in Mice

**DOI:** 10.3390/foods12061183

**Published:** 2023-03-11

**Authors:** Yali Yu, Shiyao Guan, Mengmeng Feng, Lijun Wang, Feng Gao

**Affiliations:** College of Food Science and Engineering, Jilin University, Changchun 130062, China

**Keywords:** corn germ meal, albumin peptide fractions, alcohol-induced acute liver injury, hepatoprotective activity

## Abstract

Acute alcoholic liver disease can cause serious liver damage. This study reports on the hepatoprotective effect of albumin peptide fractions from corn germ meal (MW < 1 kDa) (APF4) on acute alcohol hepatic damage in mice. In the mice model, the results indicated that APF4 at a dose of 800 mg/kg/bw could markedly boost alcohol metabolism, which was shown in the reduced duration of the loss of the righting reflex; the reduced level of blood alcohol concentration (BAC), cytochrome P450 2E1 (CYP2E1), alanine aminotransferase (ALT), aminotransferase (AST), triglycerides (TG), and malondialdehyde (MDA) (*p* < 0.01); the enhanced activity of aldehyde dehydrogenase (ALDH); and the superoxide dismutase (SOD) and glutathione (GSH) levels being increased by up to 84.02% and 193.22% (*p* < 0.01) compared to the control group. The antioxidant capability and lipid peroxidation inhibition activity of APF4 may be responsible for its protective effect against liver damage induced by alcohol. The findings suggested that APF4 had the hepatoprotective property against liver damage induced by alcohol.

## 1. Introduction

Alcohol abuse is currently a major health and socio-economic problem worldwide. Excessive alcohol intake could give rise to the injury of the liver [1]. Alcoholic liver disease (ALD) is widely regarded as the key reason for liver disease [2,3,4]. Excessive alcohol consumption has been linked to the progression of ALD to alcoholic hepatitis, liver cirrhosis, and liver cancer, and has even led to acute hepatic failure or death in some severe cases [5,6]. Therefore, it is critical for people to avoid the alcohol-induced impairment of liver function.

The liver is the main organ responsible for ethanol metabolism. Microsomal ethanol oxidase (MEOS), alcohol dehydrogenase (ADH), and catalase (CAT) are the major enzymes or enzyme systems in the liver that can catalyze the oxidation of ethanol [7,8]. These oxidative reactions produce acetaldehyde, which is then changed into acetate by an enzyme called aldehyde dehydrogenase (ALDH). Excessive drinking will exceed the liver’s detoxification capacity, which could lead to the accumulation of acetaldehyde in the blood and liver [9]. Acetaldehyde, a highly toxic substance, can result in the activation of the MEOS pathway and the production of many reactive oxygen species (ROS) [10]. The hazardous metabolite acetaldehyde, which is produced together with free radical production and the synthesis of malondialdehyde (MDA) during the metabolism of ethanol, may bind to adjacent proteins to generate MDA-acetaldehyde adducts that can cause an inflammatory response by upregulating cytokines. Additionally, the metabolism of ethanol produces the dangerous metabolite acetaldehyde, as well as free radical production and MDA creation, which would also induce an increase of the ratio of NADH/NAD^+^ (nicotinamide adenine dinucleotide), increase the probability of oxidative stress, and activate the antioxidant defense system [11]. Thus, substances with antioxidant activity could have the ability to attenuate the liver damage caused by alcohol.

Studies have revealed that peptides with bioactive properties derived from food have strong antioxidant activity and could effectively attenuate alcohol-induced liver injury. Feng et al. reported that the addition of active peptides from *Camellia vietnamensis* remarkably improved the metabolism of acute alcoholic liver injury cells [12], Cho et al. demonstrated that protein hydrolysates of mealworm could protect liver cells against ROS-induced cytotoxicity by activating cellular antioxidant systems via the Nrf2 pathway [13], and Ren et al. found that peptides purified from *Corbicula fluminea* significantly increased the viability of ethanol-injured LO2 cells [14]. It has also been reported that peptides from black soybean [15], oyster [16], and krill [17] might influence hepatoprotective efficacy by their effective antioxidant activities and anti-inflammatory properties.

Corn germ meal (CGM), an important by-product of corn deep processing, is a good resource for protein. Corn germ meal proteins are composed mainly of albumin (30%), globulin (30%), and glutelin (25%) [18]. Our previous study found that peptides (MW < 1 kDa) from corn germ meal albumin proteins displayed good free radical scavenging activity and could promote alcohol metabolism in mice [18]. Hence, the objective of this paper was to assess the hepatoprotective activity of albumin peptide fractions (MW < 1 kDa) (APF4) from corn germ meal on acute liver damage induced by alcohol. The findings of this study could be of practical guiding significance for developing a liver-protecting peptide of APF4.

## 2. Materials and Methods

### 2.1. Materials

Neutral protease was obtained from Tianjia Biotechnology Company (Changchun, China). CGM was supplied by Beidahuang Group (Changchun, China). NAD^+^, ADH, and ALDH detection kits were obtained from Ruiyong Biotechnology Company (Shanghai, China). The CYP2E1 detection kit was supplied by Eiaab Science Co., Ltd. (Wuhan, China). Commercial kits for the determination of aminotransferase (AST), alanine aminotransferase (ALT), triglycerides (TG), glutathione (GSH), malondialdehyde (MDA), superoxide dismutase (SOD), and catalase (CAT) were sourced from Nanjing Jiancheng Bioengineering Research Institute (Nanjing, China). All other chemicals and reagents used were analytical or chromatographic grade.

### 2.2. Preparation of APF4 (MW < 1 kDa)

An amount of 100 g of CGM was smashed into powder, mixed with water (1:20, *w*/*v*), and incubated for 30 min at 40 °C with continuous stirring. The solution was then centrifuged at 4 °C for 10 min at 8000 rpm, and the pH was adjusted to 4.6 with 1 M HCl. Then, the sample was centrifuged at 4 °C for 30 min at 4000 rpm. The supernatant was collected, freeze-dried by FD-1D-50 lyophilizer (Beijing, China), and stored at 4 °C for further analysis.

CGMA (1 mg/mL) was mixed with deionized water and incubated in a 100 °C water bath for 10 min. The solution’s temperature was then lowered to 51 °C, and its pH was adjusted to 6.9 with 1 M HCl. The CGMA sample was then hydrolyzed for 2 h with neutral protease (4013 U/g) and then heated in a boiling water bath for 10 min. After the hydrolysate was cooled to room temperature, it was centrifuged at 4 °C for 10 min at 8000 rpm. The supernatant was collected, freeze-dried, and stored at 4 °C.

The hydrolysate sample was fractionated by ultrafiltration membrane with a 1 kDa MW cut-off. The fraction was collected as <1 kDa (APF4) and then lyophilized for further analysis.

### 2.3. Blood Alcohol Concentration (BAC)

**Figure 1:**
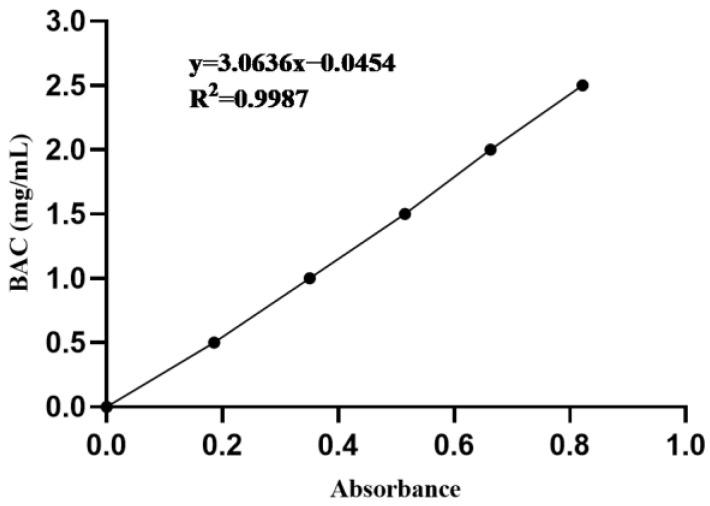
The standard curve of blood alcohol concentration (BAC).

Alcohol dehydrogenase activity (ADH) was used to determine blood alcohol concentration according to the previous study by Yu et al. [18]. The normal mice blood was coagulated with heparin to prepare the whole blood. Ethanol standard solutions were prepared with blood concentrations of 0.5, 1.0, 1.5, 2.0, and 2.5 mg/mL. The standard solution (0.25 mL) was then diluted with 4% perchloric acid (4 mL). At 4 °C, the mixture was centrifuged at 3500 rpm for 5 min. ADH-NAD^+^ solution (4.9 mL) and the supernatant (0.1 mL) were incubated for 1 h at 36 °C. The absorbance was detected at 340 nm, with absorbance as the abscissa and BAC as the ordinate to make the standard curve (Figure 1). BAC was counted according to the standard curve.

### 2.4. Animal Model to Screen the Maximum Intoxication Dose

The male Institute of Cancer Research (ICR) mice (8 weeks of age, 20 ± 2 g) were purchased from Changchun Yisi Laboratory Animal Technology Co., Ltd. (Changchun, China) and were then given 1 week to adapt before the experiments. The mice were kept in cages at 22 ± 2 °C with free access to water and food, with 12 h light–dark cycles and 60 ± 10% relative humidity.

A total of 50 mice were divided into 5 groups (10 mice for each) at random after a week of acclimatization; 50% (*v*/*v*) alcohol solution (15, 14, 13, 12, and 11 mL/kg/bw, respectively) was then administered intragastrically to the mice after a 12 h fasting. To evaluate the tolerance to alcohol in mice, Liang et al. [19] used a loss of righting reflex (LORR) experiment. After alcohol treatment, within 30 s, a mouse’s inability to right itself three times is the definition of LORR. Timing was started immediately after intragastric administration, and the number of deaths and intoxicated mice within 24 h were recorded. When LORR took place for more than 30 s, the mice were considered intoxicated.

### 2.5. The Sobering Effect of APF4

After a week of acclimatization, 60 mice were assigned to 6 groups (10 mice for each) at random: the normal control group, the alcohol control group, the positive control drug group, and APF4 in three different doses (200, 400, and 800 mg/kg/bw). The normal and alcohol control groups received saline (0.2 mL) after a 12 h fast, while the positive control drug group received 0.2 mL of 50 mg/mL/bw GSH. Then, APF4 was administered intragastrically to the APF4 groups at different concentrations. After intragastric administration, in 30 min, 12 mL/kg/bw of 50% (*v*/*v*) alcohol was given to the mice. LORR, recovery time of righting reflex, intoxication rate, and the rate of death were collected within 24 h.

### 2.6. The Dose–Response and Time–Response Relationship of APF4 on BAC after Acute Alcohol Intoxication in the Animal Model

After a week of acclimatization, 310 male ICR mice were assigned to 5 groups (60 mice for each) at random: the alcohol control group, the positive control drug group, and APF4 in three different doses (200, 400, and 800 mg/kg/bw); the remaining 10 mice were used as the blank group to make the standard curve of BAC. The following operations are the same as those described above. At 0.5 h, 1 h, 1.5 h, 2 h, 3 h, and 6 h, 10 mice were selected from each group and were sacrificed after ethanol administration. Blood samples were collected for BAC assay.

### 2.7. Determination of Liver Biochemical Parameters

The liver samples were prepared by homogenization in a saline solution (1:9, *w*/*v*) and were centrifuged at 3500 rpm for 10 min before the supernatant was collected. The liver levels of TG, AST, ALT, GSH, and MDA, as well as CAT and SOD activities, were tested by detection kits (Nanjing Jiancheng Bioengineering Research Institute, Nanjing, China). The activity of ALDH was detected using the commercial kit (Ruiyong Biotechnology Company, Shanghai, China). The CYP2E1 content was examined by the commercial kit (Eiaab Science Co., Ltd., Wuhan, China).

### 2.8. Histopathologic Observation

Fresh liver tissues were removed, fixed with 10% formaldehyde, dehydrated, and then embedded in paraffin. After the above operations, the tissues were cut into 4–5 μm sections and finally stained with standard hematoxylin and eosin (H&E) dye. An Olympus CH-2 microscope (Olympus, Tokyo, Japan) was utilized to examine the sections.

### 2.9. Statistical Analysis

All data were expressed as mean ± standard deviation (SD). One-way analysis of variance (ANOVA) followed by Duncan’s multiple range test was performed to analyze the data using the SPSS 16.0 statistical software program. Differences were considered statistically significant when *p* < 0.05.

## 3. Results

### 3.1. The Maximum Intoxication Dose

The appropriate alcohol dose for intragastric administration is very important for the acute liver damage induced by alcohol in animals. The mice would not reach the state of intoxication when administered a relatively low alcohol dose. However, a high dose might cause the death of mice due to acute gastroenteritis or abdominal distension [20].

The effects of alcohol dose by intragastric administration within 24 h on the mice of each group are shown in Table 1. The intoxication rate and the mortality rate increased with the increasing of the alcohol dose. When the mice were intragastrically administered 50% (*v*/*v*) ethanol solution (15 mL/kg/bw), all the 10 mice in the experimental group showed LORR. At the same time, five mice died and the other five mice recovered the righting reflex within 24 h of intragastric administration. The intragastric dose of ethanol (14 and 13 mL/kg/bw) resulted in a drop in the intoxication rate to 90%, and the mortality rates were 40% and 10%, respectively. The intoxication rates were 70% and 20% when the doses of ethanol were 12 and 11 mL/kg/bw, respectively; most importantly, when the doses were 12 and 11 mL/kg/bw, the mortality rate was zero. Hence, 12 mL/kg/bw was selected as the maximum intoxication dose for intragastric administration to conduct the animal model of acute alcoholic liver injury.

### 3.2. The Sobering Effect of APF4

Glutathione (GSH), abundant in living systems, is a major antioxidant, preventing all cells from free radical damage and aiding in detoxification [21,22]. GSH (50 mg/kg/bw) was used in this study as the positive control drug. Loss of righting reflex (LORR), recovery time of righting reflex, intoxication rate, and the rate of death within 24 h were used to study the sobering effect of APF4. The results of the sobering effect of APF4 are shown in Table 2. The data indicated that APF4 pretreatment could effectively decrease the intoxication rate, prolong the time of LORR, and decrease the recovery time of the righting reflex. Compared to the alcohol model group, the intoxication rates of the positive control drug group and APF4 groups were all decreased. The intoxication rate dropped to 60% when the mice were pretreated with APF4 (800 mg/kg/bw). Significant changes in extending LORR were observed in mice of the positive control drug group and the APF4 groups (400, 800 mg/kg/bw) as compared with the alcohol model group, which were 12.5 min (*p* < 0.05), 14.17 min (*p* < 0.01), and 20.67 min (*p* < 0.01), respectively. For the APF4 groups (400, 800 mg/kg/bw), the recovery times of the righting reflex were significantly reduced to 98.33 ± 40.88 min and 61.67 ± 19.09 min (*p* < 0.01). In conclusion, the effect of the low dose of APF4 (400 mg/kg/bw) on promoting alcohol metabolism was comparable to that seen in the positive control drug group, and the high-dose APF4 group (800 mg/kg/bw) was superior to the positive control drug group. The results show that taking a certain dose of APF4 in advance might prevent drunkenness to some extent and could play a positive role in sobering up.

### 3.3. Effect of APF4 on BAC

Blood alcohol concentration (BAC) is linked to the metabolism and absorption of alcohol [23]. The dose–response of APF4 on BAC after acute alcohol intoxication is shown in Table 3. The APF4 groups showed relatively lower levels of BAC as compared with the alcohol model group. At the same time of alcohol administration, the APF4 groups showed a good dose–response relationship, in which the high-dose APF4 group (800 mg/kg/bw) showed the strongest ability to reduce the BAC level (*p* < 0.01).

Table 3 also shows the time–response of APF4 on BAC level after acute alcohol intoxication. The BAC level was remarkably enhanced (*p* < 0.05) in the alcohol model group during 0.5–1.5 h. The level of BAC reached its peak at 1.5 h and then decreased significantly (*p* < 0.05). The APF4 groups, the positive control drug group, and the alcohol control group showed a consistent trend.

### 3.4. Effect of APF4 on AST, ALT, and TG Levels

**Figure 2:**
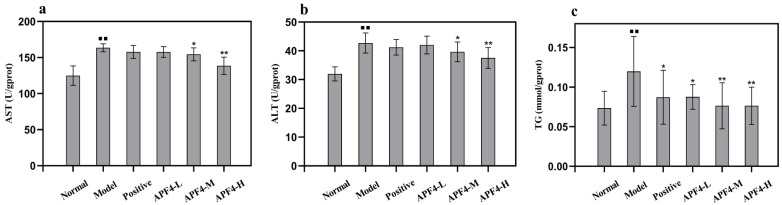
Effect of APF4 on levels of AST (**a**), ALT (**b**), and TG (**c**) in alcohol-induced liver damage mice. Values are expressed as mean ± SD, n = 10. ^⯀⯀^ *p* < 0.01 vs. the normal group, * *p* < 0.05 and ** *p* < 0.01 vs. the alcohol model group.

The results of APF4 on the AST, ALT, and TG levels in mice are shown in Figure 2. As can be seen in Figure 2a,b, AST and ALT levels from the alcohol model group were 105.47 ± 4.35 U/gprot and 36.58 ± 4.06 U/gprot, respectively, which were increased (*p* < 0.01) as compared with the normal control group, indicating that the model was successfully established. Compared with the alcohol model group, AST and ALT levels in mice from the positive control drug group and each APF4 group were all decreased. Compared to the alcohol model group, in the APF4 group (800 mg/kg/bw), significantly decreased AST and ALT levels were found (*p* < 0.01). AST and ALT levels in the liver from the APF4 groups were similar to those from the positive control drug group, indicating that APF4 had the same anti-alcohol-induced liver injury effect as GSH in vivo.

As is shown in Figure 2c, in the alcohol control group, the TG level was 0.07 ± 0.02 mmol/gprot and was increased significantly by 63.37% (*p* < 0.01) as compared with the normal control group, suggesting that alcohol administration resulted in the metabolic disorder of serum lipids in mice. However, mice pretreated with APF4 (400, 800 mg/kg/bw) had a significant reduction in serum TG level. Compared to the alcohol control group, the serum TG level in the APF4 (400 and 800 mg/kg/bw) groups decreased by 36.23% and 36.25%, respectively (*p* < 0.01). This shows that APF4 could defend the mice from acute alcoholic liver damage, and the effect of the high-dose group was the best. The results indicate that APF4 can reduce the risk of liver fat formation induced by alcohol.

### 3.5. Effect of APF4 on Alcohol-Metabolizing Enzymes, Antioxidant Enzymes, and MDA in the Liver

**Figure 3:**
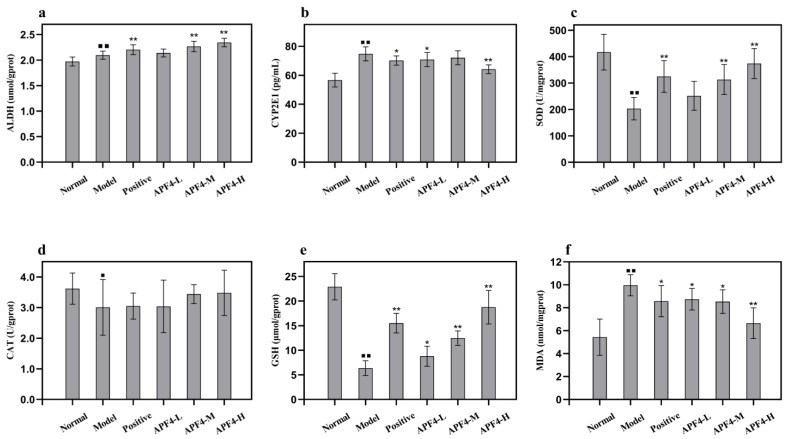
Effect of APF4 on ALDH (**a**), CYP2E1 (**b**), SOD (**c**), CAT (**d**), GSH (**e**), and MDA (**f**) activities in alcohol-induced liver damage mice. Values are expressed as mean ± SD, n = 10. ^⯀^ *p* < 0.05 and ^⯀⯀^ *p* < 0.01 vs. the normal group, * *p* < 0.05 and ** *p* < 0.01 vs. the alcohol model group.

As is shown in Figure 3a, in the normal and alcohol control groups, the levels of ALDH were 1.97 ± 0.09 μmol/gprot and 2.10 ± 0.08 μmol/gprot, respectively. Compared to the normal group, in the alcohol control group, the level of ALDH was remarkedly risen by 2.09% (*p* < 0.01), indicating that ALDH was enhanced by the activation of ethanol synthesis to metabolize the large amount of alcohol ingested by the body. APF4 at each dose group effectively increased the ALDH level as compared to the alcohol model group. In the medium- and high-dose group of APF4, the ALDH levels were significantly increased by 2.26% and 2.34% (*p* < 0.01) as compared to the alcohol control group. In the normal and alcohol control groups, the CYP2E1 levels were 56.61 ± 4.80 pg/mL and 74.76 ± 4.86 pg/mL, respectively, as shown in Figure 3b. Compared to the normal group, in the alcohol control group, the CYP2E1 level was remarkedly increased by 32.07%, indicating that the mice developed symptoms of acute alcoholism. Compared with the alcohol control group, APF4 (800 mg/kg/bw) had the most significant effect, which was a decrease of 14.19%. In the group with the high dose of APF4, the CYP2E1 level was noticeably reduced (*p* < 0.01). CYP2E1 is a major producer of alcohol-induced ROS. The decreased level of CYE2E1 indicated that the production of ROS, which is related to lipid metabolism, was restrained due to the accelerating alcohol metabolism caused by the APF4.

As is shown in Figure 3c, compared to the normal group, in the alcohol control group, there was an observable drop in the SOD activity (51.27%) (*p* < 0.01). Mice pretreated with APF4 (400, 800 mg/kg/bw) effectively increased the activity of SOD (*p* < 0.01); the most significant increase (84.02%) was seen in the high-dose APF4 group. Compared to the alcohol control group (Figure 3d), the positive control drug group and APF4 groups reduced the CAT activity. Compared to the normal group, in the alcohol control group, the GSH content was reduced (72.10%) (*p* < 0.01) and the content of MDA was increased (83.43%) (*p* < 0.01) (Figure 3e,f). The APF4 group (800 mg/kg/bw) exhibited a rise in the content of GSH (193.22%) (*p* < 0.01) and a reduction in the content of MDA (33.10%) (*p* < 0.01). These findings indicate that the antioxidant capacity and lipid peroxidation inhibitory activity of APF4 may be one possible protective mechanism of APF4 against liver injury induced by alcohol.

### 3.6. Effect of APF4 on Pathological Changes Induced by Alcohol

**Figure 4:**
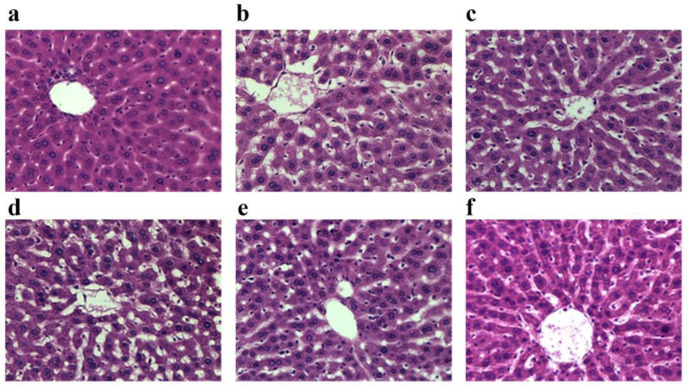
The liver histopathological sections (H&E, 200× magnification). (**a**) The normal control group; (**b**) the alcohol control group (12 mL/kg/bw); (**c**) the positive control drug group + alcohol; (**d**) APF4 (200 mg/kg/bw) + alcohol; (**e**) APF4 (400 mg/kg/bw) + alcohol; and (**f**) APF4 (800 mg/kg/bw) + alcohol.

Histopathological examinations were further carried out to confirm the hepatoprotective effect of APF4 (Figure 4). Livers from the normal control group had well-ordered hepatocytes with a normal morphology, plentiful cytoplasm, identifiable cell boundaries, a clear nucleus, and no signs of fat degeneration (Figure 4a). In contrast, the alcohol control group showed severe liver damage, which was characterized by hepatocyte necrosis, cellular degeneration, lipid droplet accumulation, and hepatocyte disarrangement (Figure 4b) compared to the normal group. In the low-dose APF4 group, compared to the alcohol control group, there were no significant changes in the liver tissue (Figure 4d). Similar to the positive control drug group (Figure 4c), in the APF4 group (400 mg/kg/bw), the injury to the central vein and hepatic cords was moderately reduced (Figure 4e). Similar to the normal group, the livers from the group with a high dose of APF4 (Figure 4f) showed restored hepatocyte morphology, vanished lipid droplets, a clear hepatocellular border, and intense plasma. These histological observations support our findings that APF4 exhibited potential liver protection.

## 4. Discussion

Alcohol abuse has become a main global health concern, resulting in significant social and economic burdens [24]. Alcohol metabolism in the human body is mainly dependent on the liver [25]. Short periods of heavy drinking can cause serious injury to the liver, and acute alcoholic liver disease is very likely to occur [26]. Currently, interest in food-derived bioactive peptides is growing because of their biological activity and high nutritional and safety characteristics [23]. Many studies in recent years have discovered the protective effect of bioactive peptides against alcohol-induced injury, such as marine collagen peptides [27], *Corbicula fluminea* protein hydrolysates [28], glycopeptide from zein [29], soybean meal peptides [30], coix seed protein hydrolysates [31], and so on. Therefore, this experiment was conducted to study the effects of albumin peptide fractions from corn germ meal (MW < 1 kDa) (APF4) on acute liver injury induced by alcohol in mice.

The alcohol dehydrogenase oxidation system, microsome alcohol oxidation system, and catalase (CAT) oxidation system are three alcohol metabolic pathways in the liver. Aldehyde dehydrogenase (ALDH) in the dehydrogenase oxidation system and CYP2E1 in the microsome alcohol oxidation system are involved with most of the alcohol metabolism, and only a small part of the alcohol intake is metabolized by the catalase (CAT) oxidation system [32]. The conversion of acetaldehyde to acetic acid is catalyzed by ALDH enzymes after alcohol is converted to acetaldehyde by ADH enzymes. CYP2E1 is a significant factor in the oxidative stress and liver damage induced by alcohol. Alcohol is metabolized and activated by CYP2E1 to more toxic and reactive products such as 1-hydroxyethyl radical and acetaldehyde [33]. In our current work, APF4 appeared to increase the activity of ALDH and decrease the CYP2E1 level in alcohol-induced mice, as compared to the alcohol control group, suggesting that APF4 had anti-alcohol effects on liver damage.

Aspartate aminotransferase (AST), found mainly in hepatocyte mitochondria, is usually used as medical clinical index for liver function examination. Alanine aminotransferase (ALT), the most specific cytosolic enzyme to the liver, is another indicator of liver damage. In nature, both ALT and AST are cytoplasmic; however, changes in liver cell membrane permeability allow these enzymes to enter the circulatory system when liver cells are damaged and/or necrotic, which results in the increasing of the concentration of ALT and AST levels in the liver [34]. Thus, increased AST and ALT levels are indicators in detecting acute and chronic liver injury. Alcohol intake can result in the accumulation of TG, which then causes hepatic steatosis. Thus, an elevated TG level is recognized as a key indicator of liver injury [35]. In our work, APF4 may have inhibited the increases of AST, ALT, and TG levels caused by alcohol, indicating that APF4 could lower the risk of alcohol-induced liver damage.

Alcohol metabolism typically leads to the production of reactive oxygen species (ROS). The production and accumulation of ROS aggravate oxidative stress in the liver, which plays a significant role in the progress of liver damage induced by alcohol [36]. MDA, the final lipid peroxidation product, is a frequent index of radical-mediated oxidative stress. Antioxidant defense systems exist in the body to protect against oxidative effects induced by ROS. SOD, CAT, and GSH are vital for the system of endogenous antioxidant defense and play a major role in metabolizing alcohol [37]. Thus, changes in the content or activity of SOD, CAT, GSH, and the hepatic MDA may be used as indirect indicators of liver damage induced by alcohol to determine the anti-oxidation status in vivo. In our study, we found that alcohol could significantly reduce the SOD and GSH activities in mice, as compared to the normal group. Once pretreated with APF4, SOD and GSH activities were restored in alcohol-induced liver injury mice. Compared to the alcohol control group, APF4 appeared to increase the CAT activity in the liver; however, the growth effect was not significant (*p* > 0.05). This might be because catalase was only required in specific circumstances [18]. APF4 also reversed the MDA increase caused by alcohol. These results indicate that APF4 could efficiently remove ROS from the liver. Our findings could shed light on new thoughts for the improvement of liver function.

## 5. Conclusions

In conclusion, this study showed that APF4 possesses the activity of facilitating alcohol metabolism and hepatoprotection in vivo. APF4 raised the acute alcohol toxicity tolerance and had a hepatoprotective effect in mice with acute alcohol-induced liver injury, as compared to the alcohol model group. The mechanisms might be related to its capability to scavenge free radicals and the suppression of lipid peroxidation. This provides a theoretical basis for the application of APF4; further studies are required to elucidate the precise mechanisms of hepatoprotection of APF4 by metabolomics and proteomics technologies.

## Figures and Tables

**Table 1:** Screening the maximum intoxication dose.

Alcohol Intake (mL/kg/bw)	Number	Intoxication Rate (%)	Mortality Rate (%)
15	10	100	50
14	10	90	40
13	10	90	10
12	10	70	0
11	10	20	0

**Table 2:** The sobering effect of APF4 in mice.

Groups	Materials	Intoxication Rate (%)	Mortality Rate (%)	Loss of Righting Reflex (min)	Recovery Time of Righting Reflex (min)
Model	Saline + Alcohol	80	0	12.00 ± 4.60	171.38 ± 47.05
Positive	GSH + Alcohol	70	0	24.50 ± 9.85 *	98.00 ± 28.92 **
APF4-200	APF4 + Alcohol	70	0	18.29 ± 8.96	143.71 ± 53.15
APF4-400	APF4 + Alcohol	60	0	26.17 ± 10.55 **	98.33 ± 40.88 **
APF4-800	APF4 + Alcohol	60	0	32.67 ± 11.71 **	61.67 ± 19.09 **

Values are expressed as mean ± SD, n = 10; GSH, glutathione; APF4, corn germ meal albumin peptides (CGMAPs) fraction (MW < 1 kDa); * *p* < 0.05 and ** *p* < 0.01 vs. the alcohol model group.

**Table 3:** The dose–response and time–response relationship of APF4 on blood alcohol concentration (BAC) after acute alcohol intoxication.

	BAC (mg/mL)				
Time (h)	Model	Positive	APF4-200	APF4-400	APF4-800
0.5	2.88 ± 0.26	2.58 ± 0.25 *	2.68 ± 0.42	2.09 ± 0.38 **	2.37 ± 0.15 **
1.0	4.20 ± 0.30	3.92 ± 0.15 *	3.99 ± 0.31	3.44 ± 0.31 **	2.71 ± 0.50 **
1.5	4.72 ± 0.35	4.50 ± 0.33	4.37 ± 0.37 *	4.10 ± 0.27 **	3.64 ± 0.26 **
2.0	4.43 ± 0.40	4.33 ± 0.20	4.23 ± 0.35	3.90 ± 0.17 **	3.10 ± 0.25 **
3.0	3.39 ± 0.29	2.62 ± 0.24 **	3.04 ± 0.15 **	2.29 ± 0.27 **	2.21 ± 0.26 **

Values are expressed as mean ± SD, n = 10; BAC, blood alcohol concentration; APF4, corn germ meal albumin peptides (CGMAPs) fraction (MW < 1 kDa); * *p* < 0.05 and ** *p* < 0.01 vs. the alcohol model group.

## Data Availability

Data sharing not applicable.
